# Recommendations to improve race identification in health records: A rapid scoping review

**DOI:** 10.1371/journal.pone.0339025

**Published:** 2025-12-29

**Authors:** Megan Chow, Arrani Senthinathan, Rasha El-Kotob, Sara J.T. Guilcher

**Affiliations:** 1 Faculty of Health Sciences, Queen’s University, Kingston, Ontario, Canada; 2 Holzschuh College of Business Administration, Niagara University, Lewiston, New York, United States of America; 3 Leslie Dan Faculty of Pharmacy, University of Toronto, Toronto, Ontario, Canada; 4 St. John’s Rehab Research Program, Sunnybrook Research Institute, Toronto, Ontario, Canada; 5 Rehabilitation Sciences Institute, Temerty Faculty of Medicine, University of Toronto, Toronto, Ontario, Canada; 6 Department of Physical Therapy, Temerty Faculty of Medicine, University of Toronto, Toronto, Ontario, Canada; Emory University, UNITED STATES OF AMERICA

## Abstract

Race is a critical variable in understanding health disparities, yet health databases lack consistent practices for identifying race. This rapid scoping review aimed to examine existing recommendations for identifying race in health databases and highlight gaps in current literature to guide future research. Following the Joanna Briggs Institute methodology and PRISMA-ScR guidelines, searches were conducted in MEDLINE, Embase, and Scopus for relevant literature published between January 2019 and February 2025. Articles were included if they addressed race identification in health databases, were available in English, had full-text access, and were peer-reviewed, knowledge syntheses, or grey literature. All articles were double screened in Covidence, and twenty-one articles were included. Descriptive thematic analysis identified five recommendation categories, including, self-identification and patient-centered practice, standardization across healthcare systems, data quality and completeness, algorithmic and predictive methods, and disaggregated data use and cross sector collaboration. There were common findings on the value of self-identification, cross-system consistency, and tools like natural language processing and imputation models. Some articles emphasized combining multiple strategies to improve system-wide practices, and overall, minimal conflicting evidence was observed. However, gaps remain in operationalizing these recommendations across various healthcare settings. Future directions should prioritize implementation-focused research and cross-jurisdictional comparisons to inform scalable, equity-driven improvements in race data practices. Ultimately, improving the consistency and accuracy of race data will enhance health equity monitoring, guide equitable resource distribution, and inform policies that better reflect the needs of racialized populations.

## Introduction

Health databases are comprehensive repositories that store patient health data collected from different healthcare encounters, including health administrative databases, electronic health records (EHRs), electronic medical records (EMRs), clinical administrative data, and health information systems [1]. Notably, EHRs and EMRs are often used interchangeably, however, EMRs typically describe records within a single healthcare organization, while EHRs are shared across multiple settings to provide comprehensive patient care [2]. In general, these databases often capture details regarding hospital admissions, diagnoses, treatments, and demographics that can serve as important tools for health system performance monitoring and research [3–5]. Capturing social determinants of health, such as race, supports equity-focused research, system performance and tailored clinical care and policy [6]. However, race data are often not collected, incomplete, inconsistently reported, or oversimplified [6]. The capacity for health databases to support equity-focused research is therefore limited and may obscure insights into systemic health disparities [6,7].

Despite being historically misunderstood as a biological concept, race is now widely accepted as a social determinant of health, with increasing attention focused on addressing its role in perpetuating health inequities [8,9]. Race often refers to categories of people who share perceived physical characteristics that are socially constructed with meaning. The classification is rooted in discrimination and social oppression to maintain hierarchies of privilege and power [8,10]. Conversely, ethnicity refers to shared cultural identity, social practices, and heritage, such as language, religion and customs [8,10]. Recognizing this difference is critical for designing equitable health interventions and policies. Unfortunately, the current literature uses the terms “race” and “ethnicity” inconsistently, which makes it challenging when designing equitable health interventions and policies. In efforts to clarify the confusion, this review focuses on the definition of "race" while reporting the authors’ original terms and instances of conflation [11,12].

Racialized populations face disproportionate health burdens that are shaped by structural and systemic inequities, including discrimination, food insecurity, and unequal access to healthcare [7]. For instance, in Canada, chronic conditions such as diabetes are 2.3 times more prevalent among South Asian adults, 1.9 times more among Black adults, and 1.8 times more among Arab and West Asian adults compared to White adults [13]. Mental health disparities are also evident, where Southeast Asian and Arab adults are less likely to rate their mental health as excellent or good, and lower life satisfaction is reported among West Asian and Black populations. Socioeconomic inequalities compound these disparities [14]. Food insecurity is 2.8 times more common among Black adults, and core housing need is over twice as high among Arab, West Asian, and Black Canadians [15]. Indigenous groups also experience some of the highest unemployment levels in Canada [16]. Discrimination within healthcare further contributes to inequities, where approximately 50% of racialized Canadians reported unfair treatment when accessing services, including being dismissed by healthcare providers, receiving less thorough assessments, or feeling disrespected during clinical encounters [17,18]. These patterns underscore the urgency of incorporating race data into health research to inform inclusive and effective interventions.

Race is increasingly understood as a fundamental variable in health research, particularly for the detection and reduction of health disparities [19]. Yet, the collection and analysis of race data into health databases remains inconsistent across countries [19]. Some health systems collect race information directly through EHRs, while others rely on surveys, geographic proxies, or algorithmic estimates based on names or locations [19,20]. These inconsistent practices differ in quality and reliability, often leading to misclassification, oversimplification, and non-comparable racial data across settings [20]. The lack of standardized frameworks, such as overgeneralized racial categories, missing data, and the absence of individual-level information, limits the ability to identify meaningful within-group differences, evaluate target interventions, and monitor longitudinal disparities in marginalized populations [21]. In health systems and research, racialized populations remain underrepresented due to variable methods of race data collection and reporting in health databases [20]. As a result, our understanding of how systemic racism and social determinants impact health outcomes for minority populations is hindered [22,23]. Strengthening the consistency and accuracy of race data across health databases is therefore essential to support equity-driven research, policy development, and system-level change.

In summary, most of the literature focused on describing disparities rather than evaluating how race information was identified or classified. To address these gaps, this rapid scoping review study aimed to 1) examine recommendations for race identification in health databases, including best practices, frameworks, guidelines, and protocols for data identification, collection, and accuracy, and 2) determine gaps in literature on recommendations for identifying race in health databases to inform further research.

## Methods

### Methodological framework

This rapid scoping review was conducted and designed based on the six-step methodology developed by Joanna Briggs Institute (JBI) Methodology for Scoping Reviews, consisting of: 1) defining the research questions and aims; 2) developing eligibility criteria for study selection; 3) performing the search strategy; 4) evidence screening and selection; 5) data extraction and analysis; and 6) the presentation of results [24]. The reported findings are aligned with the Preferred Reporting Items for Systematic Reviews and Meta-Analyses extension for scoping review (PRISMA-ScR) checklist [25]. The PRISMA-ScR checklist used in this review can be found in the supplementary information section [25]. The scoping review protocol was developed *a priori* and registered on the Open Science Framework Registries on February 3rd, 2025 (https://osf.io/yw4sd/).

### Eligibility criteria

This scoping review included articles that focused on recommendations for identifying race in health databases. Peer-reviewed studies, knowledge syntheses, and grey literature, such as dissertations and organizational articles, were included to incorporate diverse and reliable sources of information. Additionally, articles with English translations were considered to allow researchers to accurately interpret and analyze the findings.

Editorials and opinion-based sources were excluded to minimize bias and maintain focus on evidence-based recommendations. Articles published before 2019 were omitted to ensure the research reflects relevant and contemporary practices and frameworks. Sources without access to full text were excluded to ensure a comprehensive understanding of the content. Lastly, articles that focused solely on ethnicity were excluded, as the review specifically examined race data collection and reporting in health databases to address inequities tied to racial factors.

### Search strategy

Three electronic databases were searched on February 20th, 2025, including MEDLINE (Ovid), Embase (Ovid), and Scopus (Elsevier). The Ovid MEDLINE search was reviewed by lab members (MC, HZ, AS) and refined by two experienced health science librarians (AW, AR). The searches were developed using three key concepts: 1) “health databases” (e.g., health administrative databases, EMRs, digital health systems); 2) “race” (e.g., race data, racial minorities, socio-demographic data); and 3) “recommendations” (e.g., best practices, guidelines, frameworks, algorithms). Using each platform’s corresponding command languages and controlled vocabularies, the search strategies were adapted for each database when applicable. A publication year limit was applied to ensure only articles published from 2019 onward were included. The full search strategies located in the supplementary information were presented exactly as executed.

### Study selection

Articles identified from the database searches were uploaded into Covidence, an online software platform for managing reviews, on February 20th, 2025. Covidence was used for article de-duplication and screening. For consistency, three reviewers (MC, HZ, AS) preliminarily conducted a title and abstract screening of 25 articles to achieve a good interrater agreement (>80% agreement) of 88%, assessed with Microsoft Excel. Any discrepancies were resolved through virtual discussions until consensus was reached. No revisions or clarifications to the eligibility criteria were necessary, so the remaining titles and abstracts were independently double screened (MC, HZ), with any disagreements resolved by a third reviewer (AS). Next, a pilot screening of 20 full-text articles was conducted by the same reviewers (MC, HZ, AS) to ensure consistent interpretation and application of the eligibility criteria. This achieved an interrater agreement of 85% with discrepancies resolved through virtual discussions. The remaining full-text articles were independently double screened (MC, HZ), and disagreements were resolved by a third reviewer (AS).

### Data extraction and analysis

In Covidence, a structured data extraction table was formulated to guide the extraction process. Each article was double extracted by two lab members (MC, HZ) independently to enhance reliability. A third reviewer (AS) resolved any discrepancies. Extracted variables included general study information (study ID, title, authors, year of publication, journal, and country), study characteristics (objectives, design, methodology, health database examined, and racial identities analyzed), and reported recommendations for identifying race within health databases.

The extracted data were then analyzed descriptively using thematic analysis. Data were summarized by study design, health database, location, year of publication, demographic data and key findings. Next, a qualitative synthesis of the recommendations was conducted, where one lab member (MC) inductively coded the data to identify recurring themes and patterns across studies. These themes were iteratively reviewed to ensure they captured the breadth and nuances of the recommendations, allowing for a structured categorization of best practices and proposed strategies for race data identification.

## Results

### Study selection

**Fig 1:**
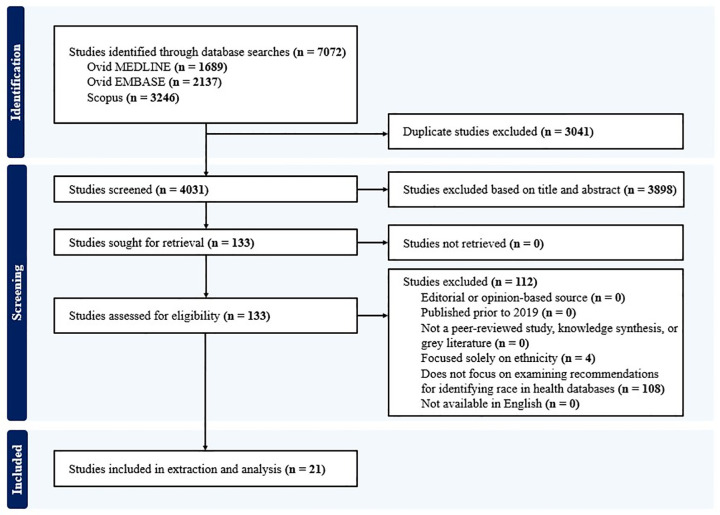
PRISMA-ScR flow diagram of articles included in the scoping review during the study identification, screening, and inclusion processes.

The database search yielded a total of 7072 articles, with 3041 duplicate studies removed, and 4031 remaining following de-duplication. At the title and abstract level of screening, 3898 articles were excluded, leaving 133 for full-text screening. Of the 133 full-text articles screened, 112 were excluded. A total of 21 studies were included in the data extraction and analysis. Fig 1 represents the PRISMA flow diagram documenting records identified, included, and excluded.

### Study characteristics

A summary of the study characteristics (author, year, country, objective, study design, health database source, and racial categories analyzed) and recommendations can be found in Table 1. Of the included studies, nearly all were conducted in North America (n = 20, 95%) [20, 26–44] in either the United States (n = 19, 90%) [20,27–44] and Canada (n = 1, 5%) [26]. The remaining study spanned multiple European countries, specifically Belgium, France and the Netherlands (n = 1, 5%) [45]. In terms of publication year, the fewest number of studies were published in 2020 (n = 1, 5%) [29] and 2025 (n = 1, 5%) [26], followed by 2021 (n = 2, 10%) [28,33], 2022 (n = 3, 15%) [37,42,43] and 2024 (n = 3, 14%) [34,38,40], 2019 (n = 4, 19%) [20,31,36,41], and the most published in 2023 (n = 7, 33%) [27,30,32,35,39,44,45].

**Table 1:** Summary of Study Characteristics and Extracted Data from Included Articles.

Authors (Year), Country	Objective	Study Design (Database Source)	Methods for Determining Race Data	Racial Identities Analyzed	Study Results	Recommendations
Abulibdeh et al. (2025) [26], Canada	Assess the content of family physicians’ EMRs and characterize the quality of the documentation of sociodemographic characteristics.	Cross sectional study (EMRs – University of Toronto Practice-Based Research Network)	Researchers assessed the completeness of 8 sociodemographic variables in EMR fields, analyzing associations with clinic, physician, and vendor characteristics using generalized linear mixed methods and machine learning.	Asian, Black, Indigenous, Latin, Middle Eastern, White, Mixed Heritage, Other	Race was one of the least documented variables (1.4% of records), with little variation across clinics indicating a consistent lack of collection.	• Standardize race and sociodemographic data collection at the point of service using structured EMR templates to document key sociodemographic variables. • Adopt universal screening protocols and incentivize data collection. • Use self-administered surveys linked to EMRs to enhance data completeness. • Use machine learning as a supplementary tool to assess EMR completeness.
Almklov et al. (2023) [27], United States	Assess whether self-reported eScreening improves the completeness and accuracy of race and ethnicity data in Veteran Affairs records, and compare self-reported data with staff-entered EHR data.	Validation study (EHRs – Veterans Health Administration Electronic Health Records from Veteran Affairs San Diego)	Researchers retrospectively compare 3 race and ethnicity data collection methods (self-reported eScreening, staff-entered EHRs, standard EHR data collection). Over 7,000 patients were analyzed for completeness and statistically validated.	American Indian or Alaska Native, Asian, Black or African American, Native Hawaiian or Other Pacific Islander, White	Self-administered eScreening and staff-entered EHR methods had lower rates of missing data and higher completeness rates than standard EHR records.	• Use self-reported data entry methods to improve data completeness over EHR entry by staff. • Include an explanation about the purpose of race data collection to promote more complete and accurate responses. • Expand response options beyond United States Office of Management and Budget framework categories to better reflect identities. • Reevaluate current race/ethnicity classification frameworks to reflect lives experiences.
Cook et al. (2021) [28], United States	Review the quality of social determinant of health data captured in EHRs for use in research and population health.	Systematic review (N/A)	Researchers analyzed 76 studies from American and Canadian health systems to determine data quality issues in social determinant of health variables. Articles were searched in PubMed and Medline, where themes were deductively synthesized and focused on completeness, plausibility, and conformance on variables.	Article did not list fixed set of racial categories but commonly included: White, Black/African American, Asian, Hispanic/Latino, Native American/Alaskan Native, Pacific Islander, Multiracial, Other, Unknown	Race was most frequently studied (53% of articles) and were primarily affected by misclassification and implausible data, especially among Hispanic, Asian, and Native American populations leading to potential.	• Implement standardized data collection protocols for race, while using self-reported data to minimize misclassification and bias. • Avoid complete case analysis to prevent biased results. • Use imputation techniques to supplement missing data and enhance accuracy. • Link and compare multiple data sources or establish auditing processes to assess accuracy. • Use validated geocoding tools and thoughtfully select patient addresses to avoid exposure misclassification.
Cusick et al. (2020) [29], United States	Improve the availability and quality of structured race data available to researchers by integrating values from multiple local sources.	Other (EHRs – EpicCare system)	Researchers supplemented missing race data by pulling on 4 local sources (inpatient EHR, billing systems, coded observations, natural language processing of clinical notes), and assessed completeness, concordance, and plausibility using a quality framework.	American Indian or Alaska Native, Asian, Black or African American, Native Hawaiian/Other Pacific Islander, White	Supplementation increased data availability from 51% to 55% adding ~142,000 patient records with no significant bias and high reliability.	• Use race data supplementation methods by integrating multiple local sources to improve race data completeness. • Apply data quality frameworks when combining datasets (e.g., Weiskopf and Weng model) to assess completeness, concordance, and plausibility of information. • Encourage standardized collection and documentation of race data at point of entry. • Use natural language processing and automatic linkage of records to capture race information from unstructured text.
Gartner et al. (2023) [30], United States	Examine methods addressing Indigenous misclassification in population-level health studies and inform future approaches to estimating Indigenous mortality/health.	Scoping review (Other – Various databases)	Researchers synthesized 97 empirical studies that addressed racial misclassification of Indigenous peoples to analyze the strengths and weaknesses of the articles’ analytical methods.	White, Black, Other Non-Indigenous, Other Nonspecific, None or comparison non applicable	Most common approaches to correct misclassification were data linkage (87%), geographic restrictions (46%), and exclusion of single-race bridging (27%). While this improved identification, it still created inconsistent race documentation, conflation of race/ethnicity/nationality, and assumptions of limited mobility.	• Use record linkage with other data sources to reduce racial misclassification. • Prioritize self-identification to ensure accurate and respectful representation. • Clarify racial categories to reflect Indigenous conceptualizations of belonging.
Hatef et al. (2019) [31], United States	Examine the availability of social and behavioural determinants of health in structured and unstructured EHRs of academic health systems.	Cross sectional study (EHRs – Multilevel academic healthcare systems across Maryland)	Researchers retrospectively analyzed EHR data to measure determinants of health data availability by comparing structured fields and unstructured text using methods such as natural language processing and structured query language.	N/A	Demographic data (e.g., race (90%)) were commonly captured, but complex variables (e.g., social connection (<1%)) were infrequent. Natural language processing and standardized data fields were valuable for supplementing missing race/social data.	• Use analytic tools (e.g., natural language processing, data mining) to extract race and sociodemographic data from unstructured EHR fields. • Implement system-wide protocols to promote standardized documentation of race.
Huang & Meyers (2023) [32], United States	Assess the validity of Medicare race and ethnicity codes by comparing Research Triangle Institute codes and a novel algorithm against self-reported data.	Validation study (Health Administrative Database – Medicare Master Beneficiary Summary File)	Researchers validated and linked datasets to compare self-reported race/ethnicity with standardized race codes to assess specificity, sensitivity, and predictive values for evaluating effects on hospitalization and readmission outcomes.	White, Black, Hispanic, Asian/Pacific Islander, American Indian/Alaska Native, Other	Codes accurately identified non-Hispanic White and Black beneficiaries but poorly captured other groups. Misclassification led to underestimation of hospitalization and readmissions which obscured differences within Hispanic subgroups by race.	• Adopt the Improved Medicare Beneficiary Survey coding algorithm, which offers better accuracy for identifying Hispanic and Indigenous populations. Use the Research Triangle Institute coding system over the Enrollment Database codes. • Revise current race/ethnicity categories to allow for more accurate reporting of multiracial identities.
Kauh et al. (2021) [33], United States	Highlight the importance of data disaggregation, challenges to implementation, and the impact of improving race and ethnicity data use in addressing health gaps.	Other (Other – Various databases/surveys)	Overview of issues in racial/ethnic data disaggregation that summarizes prior literature, policy context, and 5 empirical studies/surveys exploring subgroup disparities.	American Indian or Alaska Native, Asian, Black or African American, Native Hawaiian or Other Pacific Islander, and White	Disaggregation of racial data uncovers high amounts of variation in health, income, and social outcomes, making it essential for guiding equitable interventions and resource allocation.	• Implement policy and technical changes to enable data disaggregation. • Foster cross-sector collaboration to enhance data breadth. • Prioritize recent, self-reported data. • Move beyond minimal federal standards for racial categorizations.
Martino et al. (2024) [34], United States	Assess the accuracy of race and ethnicity data reported by home health agencies.	Validation study (Health Administrative Databases – Outcome and Assessment Information Set; Medicare Consumer Assessment of Healthcare Providers and Systems survey)	Researchers compared home health assessment data with self-reported racial data from a survey. Accuracy was statistically compared (C-statistics) with administrative datasets and Bayesian Improved Surname and Geocoding estimates.	American Indian, Alaska Native, Asian American, Native Hawaiian or Other Pacific Islander, Black, Hispanic, Multiracial, White	Bayesian Improved Surname and Geocoding estimates were most accurate, while administrative records were least accurate.	• Prioritize self-reported race and ethnicity data to enhance data accuracy. • Avoid observational methods to reduce misclassification risks. • Promote standardized practices across systems to support harmonized data collection.
Meudec et al. (2023) [45], Belgium, France, Netherlands	Examine how race and ethnicity data are used in health research, assess their role in addressing inequities, critique their conceptualization, and develop guidance for race-conscious research.	Scoping review (N/A)	Researchers analyzed completeness and reliability of country-of-birth and nationality variables in various national healthcare databases, linking them with census data to evaluate their ability to identify migrant and minority populations.	N/A	Racial identification based on administrative variables was inconsistent and limited with substantially missing data. This reflects current European databases inadequately capturing social determinants creating underestimations of health inequities.	• Promote consistency across health databases by prioritizing standardized, disaggregated, and self-identified race classifications. • Adopt race-conscious frameworks to avoid obscuring the experiences of racialized populations. • Address differences in how race and ethnicity are defined.
Polubriaginof et al. (2019) [20], United States	Evaluate the quality of race and ethnicity data in observational health databases and explore patient self-recording as a potential improvement strategy.	Validation study (Health Administrative Database – Healthcare Cost and Utilization Project; Optum Labs Data Warehouse)	Researchers assessed the completeness and accuracy of race and ethnicity data in observational databases and a New York City health system EHR to compare administrative data with self-reported survey data.	Asian, Black or African American, White, Native Hawaiian or Pacific Islander, American Indian or Alaska Native, Other, Uninformative	Race was missing for 25% of national databases and 57% of EHRs, while 86% of respondents provided meaningful race and ethnicity data when self-reporting. There was 66% discordance/misclassification between EHR and self-reported information.	• Promote self-reporting and updated race and ethnicity information to promote data completeness and accuracy. • Promote transparency and trust in data collection practices.
Samalik et al. (2023) [35], United States	Determine the accuracy of EHR-recorded race and ethnicity compared to self-report.	Validation study (EHRs – Epic (Verona, WI) at the University of Michigan)	Researchers compared EHR-recorded race and ethnicity with patient or guardian self-report across 4 pediatric specialties and used Cohen’s kappa to assess agreement.	American Indian/Alaska Native, Asian, Black or African American, Native Hawaiian/Pacific Islander, multiracial, or other	• EHR and self-report showed substantial agreement for race (0.77), however 13% of minority patients were misclassified as non-Hispanic White.	• Ensure demographic data is self-reported by patients to improve the accuracy of information. • Standardized documentation of race information.
Sholle et al. (2019) [36], United States	Develop a natural language processing method to extract race and ethnicity from clinical notes and evaluate its effectiveness compared to structured EHR data.	Validation study (EHRs – EHR at Weill Cornell Medicine)	Researchers used a rule-based natural language processing algorithm (CIREX) to extract Black and Hispanic race and ethnicity data from 4.7 million unstructured clinical notes. They compared results to structured EHR race/ethnicity data.	White, Black or African American, Indian or Alaska Native, Native Hawaiian or Pacific Islander, Multiracial, Declined	Natural language processing identified 26% more Black and 20% more Hispanic patients than structured EHR data, ultimately improving dataset completeness.	• Combine structured and unstructured data by applying natural language processing and mapping techniques to improve data completeness and accuracy.
Smith et al. (2023) [37], United States	Assess the feasibility of a centralized statewide EHR data repository for monitoring health disparities, evaluate indicator quality, and identify challenges and solutions for implementation.	Other (EHRs – Wisconsin Collaborative for Healthcare Quality EHR data)	Researchers assessed race, ethnicity, and geographic disparity indicators within a statewide centralized EHR data repository that aggregated patient-level data from 25 health systems.	American Indian or Alaska Native, Asian, Black or African American, Native Hawaiian or Other Pacific Islander, and White	Centralized harmonization improved consistency across systems. Race was available for all systems but often incomplete due to patient declining to answer, while geographic data faced harmonization problems.	• Mapping race data to national standards to improve consistency and comparability across sources. • Implement centralized data harmonization to enhance cross-system analyses. • Enhance information through natural language processing and geocoding tools.
Sojka et al. (2024) [38], United States	Assess the validity of EHR race and ethnicity data by comparing it to self-reported data.	Validation study (EHRs – Hospital EHRs in the Northeastern United States)	Researchers retrospectively compared self-reported race questionnaires from adolescents within psychiatric care against EHR-recorded data, using Cohen’s kappa to assess agreement.	White, Black, American Indian/Alaska Native, Asian, Other race, Hispanic ethnicity	Agreement between self-reported and EHR data was significant among Black (0.63) and Hispanic (0.64), moderate for White (0.54) and Asian (0.49), and limited for American Indian (0.10). 3% of EHR records included more than one race, compared to 17% of self-reports.	• Validate race and ethnicity data prior to analysis, especially when examining health outcome associations. • Collect self-reported data through multiple sources/interactions to account for the fluidity of racial and ethnic identities.
Swilley-Martinez (2023) [39], United States	Review how race and ethnicity were used and reported in epidemiologic studies and assess practices based on how they reveal or obscure systemic racism.	Systematic review (N/A)	Researchers used PRISMA-guided review methods to screen 894 articles in the American Journal of Epidemiology to extract data on how race was defined, measured, analyzed, and contextualized to assess if practices obscured systemic racism.	Black or African American, White, Hispanic, non-Hispanic Black, non-Hispanic White, Other	Most studies treated race as biological or confounding variable rather than a social construct. Determined widespread inconsistencies in collection methods and superficial use of race through broad groupings.	• Ensure transparency in how race data is collected and assigned. • Use standardized race classifications and avoid unjustified category collapsing to preserve meaningful group differences.
Weathers et al. (2024) [40], United States	Provide recommendations for improving race and ethnicity documentation at both the practice and EHR vendor levels.	Other (EHRs – Axon Registry)	Researchers reviewed literature and registry data to assess data missing in race and ethnicity fields and developed consensus-based recommendations for practices and EHR vendors to improve accuracy.	White, Black/African American, Asian, American Indian, Native Hawaiian/Pacific Islander, Other, Multiracial	29% of encounters lacked race data and 44% lacked ethnicity, limiting disparity analyses in neurological care.	• Implement standardized, patient-centered data collection frameworks through staff training and regular audits. • Enhance EHR system functionality by requiring standardized, disaggregated, and self-reported race fields.
Xue et al. (2019) [41], United States	Explore ways to improve race and ethnicity prediction in incomplete datasets using birth registry data and limited available information.	Validation study (Health Administrative Database – Connecticut Birth Registry)	Researchers used birth records (2009–2013) containing complete race and ethnicity data, to develop multinominal logistic regression models that combine Census demographics, surname probabilities, and patient-level factors to predict race and ethnicity. The model was compared against Bayesian Improved Surname Geocoding methods.	White non‐Hispanic, Black non‐Hispanic, Hispanic, and other	Model had 81% accuracy and outperformed Bayesian Improved Surname Geocoding method. Improved sensitivity for predicting Black and Hispanic race/ethnicity.	• Implement advanced predictive models that go beyond surname and geocoding to improve data accuracy. • Apply model-based imputation to fill in incomplete race data in health datasets. • Report full probability distributions from predictive models. • Tailor imputation models to local contexts and validate them against gold-standard datasets.
Yee et al. (2022) [42], United States	Assess the feasibility and implications of imputing race and ethnicity for quality and utilization measurement in Medicaid.	Cross sectional study (EHRs – Oregon Medicaid data from the Oregon Community Health Information Network)	Researchers compared self-reported race/ethnicity and Bayesian Improved Surname Geocoding/imputed race and ethnicity to estimate Hispanic-White, Black-White, and Asian-White disparities using 22 quality measures.	White, Hispanic, Black, Asian or Pacific Islander, American Indian or Alaska Native, Missing or Other	Bayesian Improved Surname Geocoding and imputation methods achieved >99% completeness and strong concordance with self-report (0.87–0.95).	• Use imputation methods (e.g., surname geocoding) when race and ethnicity data is missing. • Calibrate imputation models to local demographics and compare against gold-standard. • Incorporate full probability distributions from predictive models to reflect uncertainty.
Zavez et al. (2022) [43], United States	Improve indirect race and ethnicity imputation methods using hospitalization claims data from Connecticut.	Validation study (Health Administrative Database – Connecticut Hospital Inpatient Discharge Database)	Researchers developed a state-specific multinominal logistic regression model that incorporated first and surname dictionaries, census tract demographics, and patient-level data to impute missing race information.	Non-Hispanic White, Non-Hispanic Black, Non-Hispanic Asian and Pacific Islander, Hispanic, Non-Hispanic Other	Model achieved 85% overall accuracy and outperformed the national Census surname-based model (82% accuracy).	• Use locally tailored imputation models (e.g., region-specific name dictionaries) and incorporate both first and last names for greater accuracy. • Use probabilistic outputs in analyses.
Zhu et al. (2023) [44], United States	Provide a high-quality, publicly available resource that allows for understanding how race and ethnicity are used in clinical text.	Other (Other – Contextualized Race and Ethnicity Annotations for Clinical Text Dataset)	Researchers developed a dataset with race/ethnicity indicators and sentence-level race/ethnicity labels assigned by physician reviewers to evaluate concordance between unstructured clinical notes and structured EHR data.	Native American or Alaskan Native, Black or African American, Asian, Native Hawaiian or Other Pacific Islander, White, Not Covered, No Information	Unstructured text recovered 11.5% of missing patient race/ethnicity data in structured EHR fields, with >98% concordance with structured data.	• Use indirect estimation methods to fill in missing data in medical records when self-report is unavailable. • Use Bayesian Improved Surname Geocoding technique over other indirect methods due to stronger performance. • Utilize localized information for imputation models. • Natural language processing extraction of race from clinical notes can reduce missingness and increase granularity.

*Abbreviations*: EHR, electronic health record. EMR, electronic medical record.

All included studies employed qualitative or observational methodologies [20,26–45]. The most common study design was validation studies (n = 9, 43%) [20,27,32,34–36,38,41,43], followed by cross-sectional studies (n = 3, 14%) [26,31,42], scoping reviews (n = 2, 10%) [30,45], and systematic reviews (n = 2, 10%) [28,39]. The remaining articles were associated with other approaches (n = 5, 24%) [29,33,37,40,44], such as organizational policy statements or descriptive implementation reports. The included studies assessed a range of health databases, with the most common being EHRs (n = 9, 49%) [27,29,31,35–38,40,42], followed by health administrative databases (n = 5, 24%) [20,32,34,41,43], and EMRs (n = 1, 5%) [26]. Some studies engaged with other databases (n = 3, 14%) [30,33,44] and a few did not examine a specific database (n = 3, 14%) [28,39,45], as they focused on reviewing literature or discussing conceptual frameworks.

Approaches to identifying race and ethnicity varied across the included studies. Seven studies used the five-category classification based on the United States Office of Management and Budget framework, which includes American Indian or Alaska Native, Asian, Black or African American, Native Hawaiian or Other Pacific Islander, and White [20,32,33,35,36,39,42]. Furthermore, six studies adopted an updated seven-category framework that added Hispanic and Multiracial as distinct groups [28,29,34,39,40,44]. Notably, six studies grouped race and ethnicity into a single variable [26,31,37,41,43,45]. One study focused specifically on Indigenous populations [30], and two studies discussed identity-related concepts however did not employ a specific framework to organize race data collection (e.g., immigration status or foreign-born populations) [27,45]. These studies were retained as they provided relevant recommendations for improving race data analysis, despite discussing identity more broadly. This variation highlights ongoing challenges in standardizing race and ethnicity data collection across datasets and research contexts.

### Key Recommendations

Five overarching recommendation categories were inductively identified across the included studies: (1) Self-identification and patient-centered practice [20,27,30,33–35,38,40,45], (2) Standardization across healthcare systems [26,34,35,39,40], (3) Data quality and completeness [20,26–31,36,37,39], (4) Algorithmic and predictive methods [28,32,37,39,41–44], and (5) Disaggregated data use and cross sector collaboration [27,29,32,33,37,39,40]. The overlapping and interdisciplinary nature of efforts to improve race data in health databases was evident as numerous studies addressed more than one recommendation category. The key recommendations are summarized in Table 2, which describes each category alongside the corresponding articles and their examples.

**Table 2:** Key categories of recommendations described in included studies.

Key Recommendation	Description
Self-identification and patient-centered practice (n = 9, 43%) [20,27,30,33–35,38,40,45]	• Self-reporting as gold standard (n = 8, 38%) [20,27,30,33–35,40,45]: Several studies reported self-identification as the most accurate and complete method for collecting race data, whether through patient-completed surveys or verbal disclosure to a staff member. Contrastingly, staff-assigned inferred race and race from administrative data sources were prone to misclassification. This supports self-reporting as the preferred method for demographic data collection in healthcare settings. • Respecting the fluidity of identity (n = 2, 10%) [30,33]: Racial identity is influenced by personal, social, and political landscapes and may change over time. Patient-centered approaches acknowledge this by allowing individuals to review and update their race data as their identity evolves. Regular opportunities for revision can improve the accuracy and relevance of race data. • Race-conscious frameworks (n = 3, 14%) [27,30,45]: moving beyond vague or proxy terms (e.g., “immigrant”), to emphasize the importance of acknowledging structural inequities and using guidelines that reflect the lived realities of racialized communities in both data collection and interpretation.
Standardization across healthcare systems (n = 5, 24%) [26,34,35,39,40]	• Standardized race/ethnicity categories across systems (n = 2, 10) [35,39]: Inconsistent coding systems often fail to accurately represent certain racialized groups leading to misclassification and underrepresentation of certain groups. Studies recommend adopting improved and standardized race/ethnicity codes and ensure systems allow for multiracial and self-identified categories to support equitable health monitoring. • Universal screening protocols (n = 4, 19%) [26,34,35,40]: Implementing standardized protocols across healthcare settings can ensure that sociodemographic data is consistently collected and documented at point of care, to improve data representativeness and promoting equity across health systems.
Data quality and completeness (n = 10, 48%) [20,26–31,36,37,39]	• Using multiple data sources and mapping data (n = 5, 24%) [26,28–30,36]: Combining data from multiple sources (e.g., electronic health records, administrative records, birth certificates), can improve completeness, reduce misclassification, and enable more accurate race and ethnicity identification. Centralized data mapping and harmonization across systems help standardize race variables and reduce inconsistencies in documentation practices. • Natural language processing and other analytic tools (n = 4, 19%) [26,31,36,37]: These tools can extract race and ethnicity information from unstructured text, supplementing structured fields and enhancing identification accuracy, particularly in cases with incomplete data. • Transparency in the source of data (n = 3, 14%) [20,27,29]: Studies suggest health systems should clearly report how race and ethnicity data was collected and who assigned the classification to ensure transparency, reproducibility, and accountability in data use.
Algorithmic and predictive methods (n = 8, 38%) [28,32,37,39,41–44]	• Imputation techniques (n = 7, 33%) [28,32,37,41–45]: Imputation through tools like Bayesian Improved Surname Geocoding can supplement missing race and ethnicity data by using proxies such as surnames and geolocation. Additionally, enhanced models should incorporate additional variables (e.g., income, age) to further improve accuracy and help reduce bias from incomplete datasets. • Local tailoring and validation (n = 4, 19%) [41–44]: Imputation models should be calibrated to the specific geographic or population context using local data sources and validated against gold-standard self-reported information to ensure reliability in race classification. • Probabilistic approaches for uncertainty (n = 5, 24%) [28,39,41–43]: Models should report the full probability distribution across racial/ethnic groups to better reflect uncertainty, avoid oversimplification, and improve the validity of downstream analysis.
Disaggregated data use and cross sector collaboration (n = 7, 33%) [27,29,32,33,37,39,40]	• Disaggregated data and definitions (n = 5, 24%) [27,32,33,39,40]: Broad racial and ethnic categories often mask meaningful subgroup differences and perpetuate misclassification. Thus, using disaggregated data and clear self-identified definitions helps ensure that the diversity within racialized groups is recognized and disparities are accurately captured. • Collaboration across systems and sectors (n = 3, 14%) [29,33,37]: Recommend coordinated efforts among health systems, policymakers, and data submitters to support sustainable improvements.

## Discussion

Many important findings were identified in this rapid scoping review. First, self-identification and person-centered practices were described as the gold standard for race data collection, emphasizing patient autonomy and lived experiences [20,27,30,33–35,38,40,45]. Notably, many articles emphasized improvement to race data collection at the point of care [20,26,27,33,34,38,40], such as self-reported patient intake forms or staff-administered surveys, rather than methods that refine or supplement race data after it had been recorded. Second, multiple studies highlighted the importance of transparency in how data are sourced and reported, particularly in distinguishing between self-reported, inferred, or algorithmically imputed data [20,27,39]. Third, studies identified standardization across systems to be essential for having consistent categorization of race and enabling reliable comparisons across databases [26,34,35,39,40]. Finally, strategies to improve data completeness, such as the use of supplementing sources and predictive tools, were proposed to enhance existing datasets when self-reported data were not available [26,28,41–44]. Altogether, these findings highlight opportunities to strengthen race data practices in healthcare and bring up persistent gaps in implementation, standardization, and health research.

Self-identification was the most widely supported and validated method for collecting race data in health systems, but real-world implementation challenges remain an issue [20,26,27,30,33,34,38,40,45]. It is grounded on the premise that individuals are best positioned to define and express their own identities [26]. Several studies highlighted strategies to operationalize self-identification [26,27,30,40], such as embedding self-reported surveys into EMRs [27], with reported response validity ranging from 84% to 100% in primary care settings [45]. Almklov et al. [27] demonstrated that electronic self-reporting tools outperformed both standard EHR documentation and staff-entered race data in terms of completeness and accuracy. Patient-facing tools, such as pre-visit portals or kiosk check-ins, were also proposed for clinical implementation, allowing individuals to directly record and update their demographic information, enhancing data quality and patient control [26,34]. Self-identification would also enhance the validity of race data by reducing common misclassification errors in observational or administrative databases, while also supporting more equitable care by centering patient voices and lived experiences [20,26,27,30,34,38,40]. This shift from extractive to patient-informed data practices may foster trust and align with principles of cultural safety.

Despite these strengths, policy, resource availability, and ethical challenges continue to limit the uptake of widespread implementation. In terms of policy, the absence of national or institutional mandates for standardized race data collection leads to inconsistent adoption [46]. Resource constraints, such as staff training or digital infrastructure, further complicate routine collection within already burdened clinical settings [46]. Ethically, concerns regarding data privacy and potential misuse create hesitancy within institutions and among patients. For instance, higher rates of “decline to answer” were observed when demographic questions lacked clarity or sensitivity, or when patients were unsure how their information would be used [27]. Future directions should explore how to frame race-related self-reporting questions to encourage disclosure, particularly among populations that are hesitant to self-identify. Also, although many studies emphasized the importance of self-identification and proposed techniques for improved data collection, few evaluated their implementation in real-world healthcare settings. Most recommendations remained conceptual, offering limited insight into feasibility, cost, equity impact, and sustainability of this intervention [30,33–35,40,45]. Further research should assess these suggestions based on patient engagement outcomes and determine necessary supports to translate these approaches into routine practice.

Another key finding from our review was the need for transparency in how race data were sourced, recorded and interpreted [20,27,39]. Swilley-Martinez et al. [39] emphasized that researchers and health systems should clearly document if race information was self-reported, observed, or algorithmically assigned, and record who made the classification. This level of documentation would strengthen the credibility of race data and ensure that downstream research and policy decisions were based on credible, well-understood sources [27]. Despite these advantages, few health systems consistently tracked or reported how race data were collected, and such information was often overlooked in research publications and datasets [46]. This introduced uncertainty, limited comparability, and made it difficult to determine whether observed disparities reflected real differences or were artifacts of data collection methods [46]. Therefore, future research should focus on developing clear standards for disclosing race data sources and examining how different data collection techniques may influence the interpretation of race-based analyses in health equity research. Establishing greater transparency will assist in laying the groundwork for standardized race data practices that are credible across healthcare systems.

Our findings highlighted the need for standardized frameworks for race data collection to improve comparability, accuracy, and equity across healthcare settings and databases [32,34,35,39]. Inconsistent definitions and classification schemes were noted as common barriers to accurately identifying and comparing race data, particularly when conducting analyses across institutions or national datasets [32]. Standardization was suggested to improve data interoperability, reduce data cleaning, and enable large-scale equity analyses within health systems. For instance, Huang et al. [32] evaluated Medicare administrative data and determined significant variability in the accuracy of commonly used coding systems depending on the racial group being identified. Thus, if racial groups were organized in the same manner, health databases could consistently produce demographic information that is comparable across sources. This could augment the ability to monitor disparities, allocate resources, and inform equitable policy responses. However, it is important to consider limitations that arise with race data standardization. Implementing harmonized systems requires extensive coordination between stakeholders such as government agencies, health system leaders, and EHR vendors, which may be resource intensive [37]. Future research should investigate strategies that account for institutional readiness, stakeholder alignment, and policy supports needed to adopt standardized data practices in differing health system contexts. Additionally, overly broad and outdated categories could perpetuate exclusion and obscure meaningful differences in health outcomes, such as masking subgroup variation or collapsing multiracial identities [26]. Further studies should explore frameworks for expanding racial categories to reflect evolving identities and capture within-group diversity.

Finally, our review identified the use of supplementary and predictive models as a strategy to improve the completeness of race data where self-reported data were incomplete or missing [26,28,41–44]. This included data linkage techniques, natural language processing, and algorithmic imputations such as the Bayesian Improved Surname Geocoding model. The Bayesian Improved Surname Geocoding model was the most mentioned method and estimates a patient’s race based on their last name and geocoded address [26,28,42]. This was shown to reduce missing data and enhance the reliability of race-based categorization in large datasets [28,42,44]. Some studies explored advanced or locally tailored imputation models that incorporated other factors like age, income, or household structure to improve accuracy [41,43]. To display uncertainty, probabilistic rather than fixed categorical assignments were recommended by multiple sources [28,41–44]. For instance, reporting a patient as 70% Black, 20% Hispanic, and 10% White to provide a more nuanced analysis with room for error [41,42]. Ultimately, this might improve the utility of health datasets that lack self-identified race information and allow an increased sample size of data. However, an important consideration to note was that predictive models relied on assumptions of factors (i.e., name, location, neighbourhood composition) that may not reflect an individual's lived racial identity [41]. This could unintentionally reinforce systematic biases, especially without any data validation or transparency. Ethical concerns such as lack of consent, limited interpretability, and potential misuse in policy or research contexts should also be considered. Future directions include exploring how advancements in artificial intelligence and natural language processing could improve contextual understanding of unstructured clinical notes, enhance data linkage, and automate validation processes to reduce biases and strengthen accuracy in race data identification. Future research should continue to focus on validating these imputation models against self-reported data, identifying methods for local tailoring, and creating ethical standards for probabilistic reporting to ensure these tools are used responsibly. As predictive approaches evolve, ensuring their ethical applications will be essential for advancing the quality of research and equity-informed health policies.

While most studies presented similar recommendations, a few presented conflicting perspectives regarding feasibility and data validity. Specifically, some authors questioned whether standardized categories risk oversimplifying complex identities [20,33,38], while others argued that uniform classification is necessary for comparability across databases [34,37,39]. For instance, certain studies recommended consolidating racial categories to align with United States national standards, such as the Office of Management and Budget standardized codes for comparability [26,34,35,39], while others emphasized the need to expand definitions or disaggregate data to better capture within-group differences and nuanced identities [29,33,37]. Furthermore, while some articles viewed algorithmic approaches as practical solutions for missing data [32,36,42–44], others cautioned that they would perpetuate biases or obscure inequities [26,40,41]. These contradictions demonstrated tension in the field regarding precise data and its comparability, as well as inconsistencies in innovation and ethics. Future research should aim to explore these perspectives by integrating both patient-centered and system level approaches, which manage data accuracy along with lived experience.

Overall, these findings reflected both advancements and ongoing challenges when collecting and reporting race data in health systems. Notably, of the 21 articles identified, most were based in the United States, limiting the global applicability of the recommendations due to differences in health systems, race categorizations, and data governance structures across diverse regions. Therefore, while current research provides a constructive foundation, the small number of applicable studies relative to the broad scope of the review highlights an important area for further research. Strengthening race data practices might enable a more accurate understanding of racial disparities in healthcare use and outcomes, which may guide more effective, equity-informed interventions and policy decisions.

### Study strengths and limitations

A notable strength of this review was the comprehensiveness of the search strategy, which included interdisciplinary sources to capture diverse methodologies and perspectives on race data identification. Nevertheless, there were a few study limitations. While the database search was extensive, the review was limited to studies published between January 2019 and February 2025. This may have omitted earlier foundational work or longstanding recommendations that are still relevant today. Additionally, restricting eligibility criteria to only include English studies may have introduced selection bias by potentially excluding important findings in other languages. The review also focused specifically on race rather than ethnicity, which may have excluded studies examining broader constructs of identity and social categorization, thus potentially influencing population health outcomes. Furthermore, the quality of the included studies and their recommendations were not assessed, as critical appraisal is not typically required for scoping reviews [24]. As a result, the relative strength and reliability of individual strategies could not be evaluated.

## Conclusion

This scoping review identified current recommendations for improving the identification of race within health databases, revealing five key thematic areas: self-identification and patient-centered practices, standardization across healthcare systems, data quality and completeness, algorithmic and predictive methods, and equity-oriented and disaggregated data use. While promising strategies exist, implementation remains inconsistent, and gaps such as a lack of global applicability, limited critical appraisal, and minimal focus on real-world feasibility highlight the need for further research. Strengthening race data identification is essential not only for improving data accuracy but also for supporting equity-driven research, policy, and health system transformation. Continued efforts to refine and operationalize these recommendations are crucial to advancing more inclusive healthcare systems.

## Supporting information

S1 File PRISMA-ScR Checklist. (DOCX)

S2 File Search strategy used in MEDLINE (Ovid), Embase (Ovid), and Scopus (Elsevier). (DOCX)
