# Supplementary material for: Recommendations to improve race identification in health records: A rapid scoping review
Source: PLoS One. 2025 Dec 29;20(12):e0339025. doi: 10.1371/journal.pone.0339025 (PMC12747332; doi:10.1371/journal.pone.0339025)
Supplement: S2 File — (DOCX) [file pone.0339025.s002.docx]

Search Strategy – Ovid MEDLINE ran on February 20th, 2025

| **#** | **Search Term** | **Results** |
| --- | --- | --- |
| 1 | Racial Group/ or exp "Ethnic and Racial Minorities"/ | 28573 |
| 2 | race.tw,kf | 159869 |
| 3 | 1 or 2 | 177233 |
| 4 | Electronic health records/ or “Medical Records Systems, Computerized”/ or health smart cards/ or health information systems/ or medical informatics applications/ or database management systems/ or “databases as topic”/ or routinely collected health data/ | 69099 |
| 5 | (Health or medical) ADJ3 (record* or data?base* or smart?card* or data or “informati* system*” or “informati* application*” or “management system*”).tw,kf | 327464 |
| 6 | 4 or 5 | 367171 |
| 7 | “validation studies as topic”/ or validation study/ or intersectional framework/ | 118112 |
| 8 | (standardized approach* or best practice* or guideline* or recommend* or suggestion* or validati* or moderniz* or algorithm* or address* or availab*).ti,ab. | 4224092 |
| 9 | exp consensus/ | 24650 |
| 10 | exp consensus development conference/ | 13015 |
| 11 | exp consensus development conferences as topic/ | 3014 |
| 12 | exp guideline/ | 40146 |
| 13 | guidelines as topic/ | 42488 |
| 14 | exp practice guideline/ | 33084 |
| 15 | practice guidelines as topic/ | 131394 |
| 16 | health planning guidelines/ | 4169 |
| 17 | (guideline or practice guideline or consensus development conference or consensus development conference).pt. | 50014 |
| 18 | (position statement* or policy statement* or practice parameter* or best practice*).ti,ab,kf. | 54062 |
| 19 | (standards or guideline or guidelines).ti,kf. | 149762 |
| 20 | ((practice or treatment* or clinical) adj guideline*).ab. | 60860 |
| 21 | (CPG or CPGs).ti. | 6737 |
| 22 | consensus*.ti,kf. | 40297 |
| 23 | consensus*.ab. /freq=2 | 39978 |
| 24 | ((critical or clinical or practice) adj2 (path or paths or pathway or pathways or protocol*)).ti,ab,kf. | 30754 |
| 25 | recommendat*.ti,kf. or guideline recommendation*.ab. | 66275 |
| 26 | (algorithm* adj2 (screening or examination or test or tested or testing or assessment* or diagnosis or diagnoses or diagnosed or diagnosing)).ti,ab,kf. | 11579 |
| 27 | (guideline* or standards or consensus* or recommendat*).au. | 11 |
| 28 | (guideline* or standards or consensus* or recommendat*).cn. | 602 |
| 29 | or/7-28 | 4447737 |
| 30 | 3 and 6 and 29 | 2819 |
| 31 | exp consensus development conference/ or exp consensus development conferences as topic/ or congresses as topic/ or preprint/ or (conference* or preprint*).ti,ab,pt. | 164688 |
| 32 | limit 30 to yr="2019 -Current" | 1719 |
| 33 | 32 not 31 | 1689 |

Search Strategy – Ovid Embase ran on February 20th, 2025

| **#** | **Search Term** | **Results** |
| --- | --- | --- |
| 1 | ancestry group/ or ethnic group/ or racial identity/ | 101047 |
| 2 | race.tw,kf | 257281 |
| 3 | 1 or 2 | 343689 |
| 4 | electronic health record/ or electronic medical record system/ or smart card/ or  medical information system/ or medical informatics/ or database management system/ or data base**/** or routinely collected health data/ | 379674 |
| 5 | (Health or medical) ADJ3 (record* or data?base* or smart?card* or data or “informati* system*” or “informati* application*” or “management system*”).tw,kf | 509737 |
| 6 | 4 or 5 | 812774 |
| 7 | validation study/ or intersectional framework/ | 118897 |
| 8 | (standardized approach* or best practice* or guideline* or recommend* or suggestion* or validati* or moderniz* or algorithm* or address* or availab*).ti,ab. | 5841322 |
| 9 | exp consensus/ | 110961 |
| 10 | exp consensus development/ | 30367 |
| 11 | exp practice guideline/ | 785488 |
| 12 | health planning guidelines/ | 118186 |
| 13 | (position statement* or policy statement* or practice parameter* or best practice*).ti,ab,kf. | 76921 |
| 14 | (standards or guideline or guidelines).ti,kf. | 204512 |
| 15 | ((practice or treatment* or clinical) adj guideline*).ab. | 92175 |
| 16 | (CPG or CPGs).ti. | 8086 |
| 17 | consensus*.ti,kf. | 50113 |
| 18 | consensus*.ab. /freq=2 | 52845 |
| 19 | ((critical or clinical or practice) adj2 (path or paths or pathway or pathways or protocol*)).ti,ab,kf. | 46679 |
| 20 | recommendat*.ti,kf. or guideline recommendation*.ab. | 85225 |
| 21 | (algorithm* adj2 (screening or examination or test or tested or testing or assessment* or diagnosis or diagnoses or diagnosed or diagnosing)).ti,ab,kf. | 16263 |
| 22 | (guideline* or standards or consensus* or recommendat*).au. | 28 |
| 23 | or/7-22 | 6432839 |
| 24 | 3 and 6 and 23 | 9552 |
| 25 | exp consensus development conference/ or exp consensus development  conferences as topic/ or congresses as topic/ or preprint/ or (conference* or preprint*).ti,ab,pt. | 6677165 |
| 26 | limit 24 to yr="2019 -Current" | 4892 |
| 27 | 26 not 25 | 2137 |

Search Strategy – Scopus ran on February 20th, 2025

| **#** | **Search Term** | **Results** |
| --- | --- | --- |
| 1 | ( TITLE-ABS-KEY ( race ) AND TITLE-ABS-KEY ( "health administrative database*" OR "electronic health record*" OR "electronic medical record*" OR "digital health system*" OR ( "digital health" W/4 "information system*" ) OR "digital health system*" OR "health information system*" OR "diagnostic code*" OR "diagnosis code*" ) OR TITLE-ABS-KEY ( ( health OR medical ) W/3 ( record* OR data?base* OR smart?card* OR data OR "informati* system*" OR "informati* application*" OR "management system*" ) ) AND TITLE-ABS-KEY ( "standardized approach*" OR "best practice*" OR guideline* OR recommend* OR suggestion* OR validati* OR moderniz* OR algorithm* OR address* OR availab* ) OR TITLE-ABS-KEY ( "position statement*" OR "policy statement*" OR "practice parameter*" OR "best practice*" ) OR TITLE-ABS-KEY ( standards OR guideline OR guidelines ) OR ABS ( ( ( practice OR treatment* OR clinical ) W/0 guideline* ) ) OR TITLE ( cpg OR cpgs ) OR TITLE-ABS-KEY ( consensus* ) OR TITLE-ABS- KEY ( ( ( critical OR clinical OR practice ) W/2 ( path OR paths OR pathway OR pathways OR protocol* ) ) ) OR TITLE-ABS-KEY ( recommendat* ) OR ABS ( guideline AND recommendation* ) OR TITLE-ABS-KEY ( ( algorithm* W/2 ( screening OR examination OR test OR tested OR testing OR assessment* OR diagnosis OR diagnoses OR diagnosed OR diagnosing ) ) ) OR TITLE-ABS-KEY ( guideline* OR standards OR consensus* OR  recommendat* ) ) AND PUBYEAR > 2018 AND PUBYEAR < 2026 | 3246 |
